# LCCL protein complex formation in *Plasmodium* is critically dependent on LAP1

**DOI:** 10.1016/j.molbiopara.2017.04.005

**Published:** 2017-06

**Authors:** Annie Z. Tremp, Vikram Sharma, Victoria Carter, Edwin Lasonder, Johannes T. Dessens

**Affiliations:** a Pathogen Molecular Biology Department, Faculty of Infectious and Tropical Diseases, London School of Hygiene & Tropical Medicine, Keppel Street, London WC1E 7HT, UK; b School of Biomedical and Healthcare Sciences, Plymouth University, Drake Circus, Plymouth PL4 8AA, UK

**Keywords:** LCCL, LC/MS/MS, Crystalloid organelle, Transmission blockade

## Abstract

Successful sporogony of *Plasmodium berghei* in vector mosquitoes requires expression of a family of six modular proteins named LCCL lectin domain adhesive-like proteins (LAPs). The LAPs share a subcellular localization in the crystalloid, a unique parasite organelle that forms during ookinete development. Here, LAP interactions in *P. berghei* were studied using a series of parasite lines stably expressing reporter-tagged LAPs combined with affinity purification and high accuracy label free quantitative mass spectrometry. Our results show that abundant complexes containing LAP1, LAP2 and LAP3 are formed in gametocytes through high avidity interactions. Following fertilization, LAP4, LAP5 and LAP6 are recruited to this complex, a process that is facilitated by LAP1 chiefly through its scavenger receptor cysteine-rich modules. These collective findings provide new insight into the temporal and molecular dynamics of protein complex formation that lead up to, and are required for, crystalloid biogenesis and downstream sporozoite transmission of malaria parasites.

## Highlights

• The six LAPs in *Plasmodium berghei* form a protein complex in ookinetes.
• Complex formation begins in macrogametocytes with LAP1, LAP2 and LAP3 interactions.
• LAP1 plays a key role in recruitment of LAP4, LAP5 and LAP6 to the complex.
• The LAP complex displays different strength interactions between components.

## Graphical abstract

**Figure:**
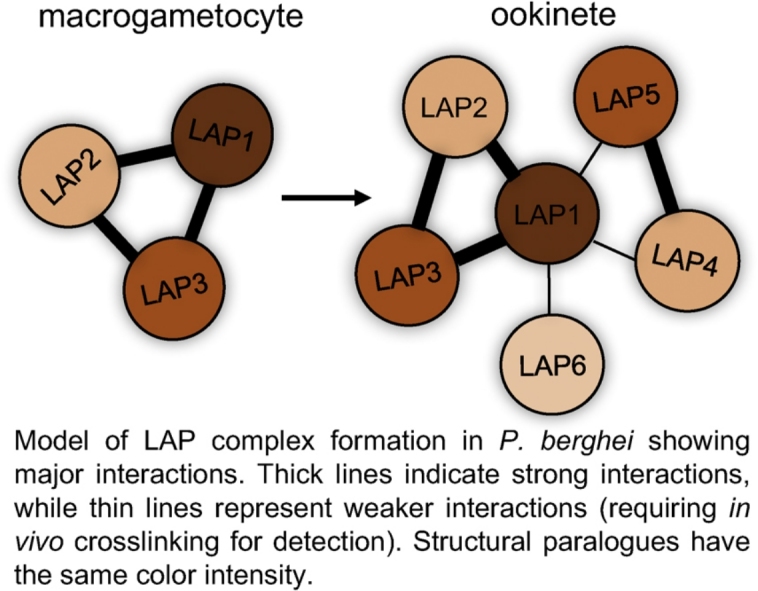

LCCL proteins form a family of unique modular proteins restricted to apicomplexan parasites [1]. The proteins obtained their name from possessing one or more copies of the ‘LCCL’ domain, a conserved protein module that was first identified in the founding proteins ***L****imulus* clotting factor **C**; cochlear protein **C**och-5b2; and lung gestation protein **L**gl1 [2]. Six LCCL protein family members have been identified in *Plasmodium*, which in *P. berghei* are mostly referred to as LCCL lectin adhesive-like protein (LAP) 1–6 [3] (Fig. 1). They have a complex and unique architecture typified by possessing multiple domains implicated in protein, lipid and carbohydrate binding [1], [4] (Fig. 1). LAP5 does not possess a predicted LCCL module and is included in the family by virtue of its otherwise similar structure to LAP3 (Fig. 1). Disruption of *lap* genes in *P. berghei*, either individually or in pairs, gives rise to very similar loss-of-function phenotypes characterized by a failure of the oocyst to produce infective sporozoites [5], [6], [7], [8], [9], [10].

**Fig. 1:**
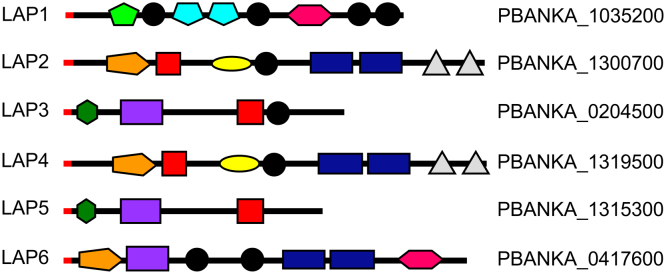
Schematic diagram of *Plasmodium* LAP1-LAP6 (PlasmoDB IDs shown on the right hand side). All proteins possess a predicted N-terminal ER signal peptide (red). A variety of modules are shown with significant homologies to known protein domains. Black: *Limulus* coagulation factor C, Coch-5b2 and Lgl1 (LCCL) domain (Pfam03815, Smart00603); Light green: Polycystin-1, Lipoxygenase, Alpha-Toxin (PLAT) domain (Pfam01477, Smart00308); Light blue: scavenger receptor cysteine-rich (SRCR) domain (Pfam00530, Smart00202); Pink: pentaxin (PTX)/Laminin-G domain (Pfam00354, Smart00159); Orange: ricin-type beta trefoil lectin domain (Pfam00161, Smart00458); Red: coagulation factor 5/8 carboxy-terminal/discoidin domain (Pfam00754, Smart00231); Yellow: fibrillar collagen (COLFI) carboxy-terminal domain (Pfam01410, Smart00038); Dark blue: Levanase-like domain; Purple: anthrax protective antigen domain (Pfam07691); Dark green: fibronectin type II domain (Pfam00040, Smart00059); Grey: apicomplexan-specific cysteine-rich domain. (For interpretation of the references to color in this figure legend, the reader is referred to the web version of this article.)

A significant advance in our understanding of the LAPs came with the discovery that they are targeted to the crystalloids and are required for crystalloid formation [5], [10], [11]. First described in 1962 [12], crystalloids are transient subcellular organelles that are implicated in malaria transmission by virtue of their exclusive presence in ookinetes and young oocysts (reviewed in [13]). The organelles are conserved in human, monkey, rodent and bird malaria species, and they appear in electron microscopy as clusters of small vesicles [14]. Whilst *P. berghei* ookinetes contain on average two crystalloids, only a single large crystalloid is found in the oocyst indicating that crystalloid biogenesis completes after ookinete-to-oocyst transition [10]. The inability to form crystalloids appears to be a shared feature of LAP knockout parasites, as is their inability to form sporozoites, thus providing a functional link between crystalloid formation and successful sporogonic development. A mutant parasite line expressing LAP3 lacking its LCCL domain and turning it into a LAP5-like protein was shown to have delayed crystalloid formation [10], further pointing to the involvement of the LAPs in crystalloid genesis.

The similar loss-of-function phenotypes of the LAPs in *P. berghei* suggest that they operate as a protein complex. Indeed, LAP orthologues in the human malaria parasite *P. falciparum* were shown to co-immunoprecipitate with specific antibodies, supporting formation of a LAP complex [15]. Furthermore, *in vitro* binding assays with recombinant, bacterially expressed proteins corresponding to various LAP portions identified putative interactions between LAP1 and all other LAPs except LAP5; between LAP2 and LAP3; and between LAP2 and LAP5 in *P. falciparum* [15]. In this paper we report a complementary *in vivo* approach to investigate LAP interactions in the rodent malaria parasite *P. berghei,* using a series of existing and newly generated genetically modified parasite lines stably expressing LAPs fused to green fluorescent protein (GFP), combined with GFP affinity purification and label-free quantitative mass spectrometry (LFQ MS).

We began by testing whether we could successfully pull down GFP-tagged LAPs from purified parasites with magnetic beads conjugated to anti-GFP antibodies (see supplemental Materials and Methods section). Gametocyte pull down samples of parasite line LAP3/GFP that expresses LAP3 fused to a carboxy-terminal GFP [11] were subjected to SDS-PAGE alongside corresponding samples from wild-type parasites, followed by protein staining (Fig. 2A). This visualized several bands specific to the LAP3/GFP sample, one of which corresponded to the target protein (LAP3::GFP) as demonstrated by western blot using anti-GFP antibodies (Fig. 2B). These results indicated that the anti-GFP antibody-bound magnetic microbeads are successful in isolating the GFP-tagged target protein as well as proteins bound to it. Indeed, subsequent MS-based proteomic analysis (see supplemental Materials and Methods section) revealed that the GFP pull-down samples harvested from the purified LAP3/GFP gametocytes reproducibly contained LAP1, LAP2 and LAP3 as the most abundant parasite proteins (Table 1 and Supplemental Table S1). This is consistent with the reported protein expression of LAP1, LAP2 and LAP3 in *P. berghei* macrogametocytes [5], [11]. Pull down from purified LAP3/GFP ookinetes, which express the full LAP repertoire [16], gave the same result, as did equivalent pull down samples from parasite line LAP1/GFP (originally called PbSR/EGFP [5]) (Tables 1, S1). As expected, LAPs were not pulled down from LAP3-KO or LAP1-KO parasites by the same method (Tables 1, S1), providing further evidence that the pull downs are specific. These collective results indicate that LAP1, LAP2 and LAP3 have high avidity interactions with each other, but not with the other LAPs.

**Fig. 2:**
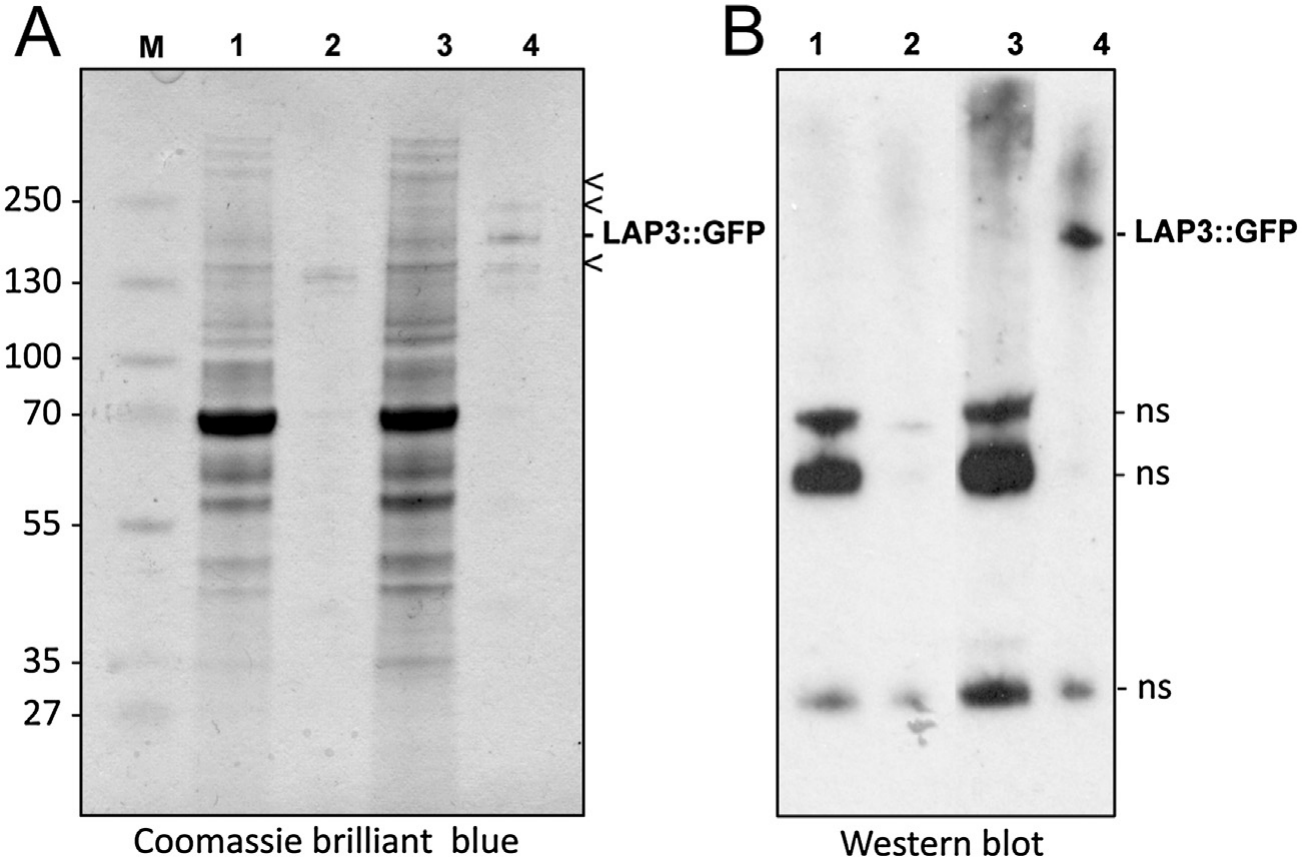
Immunoaffinity pull-down of LAP complexes from *P. berghei* gametocytes with anti-GFP antibody-coated magnetic beads. **A**: Coomassie brilliant blue staining shows specific pull down of the LAP3:GFP target protein and several other proteins (arrow heads). Lanes 1 + 2: wildtype; lanes 3 + 4; LAP3/GFP; lanes 1 + 3: before pull down; lanes 2 + 4: after pull down. **B**: Western blot using anti-GFP antibodies shows enrichment of the LAP3:GFP target protein. Nonspecific (ns) antibody binding is indicated. Molecular weight markers in kDa are shown (M).

**Table 1:** Relative abundance of LAPs in GFP pull down samples from *Plasmodium berghei* parasite lines.

Parasite line^a^	gametocyte	ookinete	crosslinked	Relative percentage LFQ intensity (number of unique peptides)					
				LAP1	LAP2	LAP3	LAP4	LAP5	LAP6
LAP3/GFP^b^	+			229 (45)	236 (55)	100 (35)	0 (0)	0 (0)	0 (0)
LAP3/GFP	+			277 (51)	93 (46)	100 (33)	0 (0)	0 (0)	0 (0)
LAP3-KO^3^	+			0 (0)	0 (0)	0 (0)	0 (0)	0 (0)	0 (0)
LAP3/GFP		+		249 (52)	261 (69)	100 (43)	0.2 (2)	0 (0)	0 (0)
LAP3/GFP		+		452 (52)	439 (73)	100 (41)	0.6 (3)	0 (1)	0 (0)
LAP3-KO		+		0 (1)	0 (2)	0 (1)	0 (0)	0 (0)	0 (0)
LAP1/GFP^d^		+		1087 (43)	316 (18)	100 (12)	0 (0)	0 (0)	0 (0)
LAP1-KO^e^		+		0 (0)	0 (0)	0 (0)	0 (0)	0 (0)	0 (0)
									
LAP4/GFP^f^		+		0 (0)	0 (0)	0 (0)	100 (14)	169 (11)	1.2 (3)
LAP4/GFP		+		0 (0)	0 (0)	0 (0)	100 (8)	18 (7)	0 (0)
LAP5/GFP^f^		+		0 (0)	0 (0)	0 (0)	652 (17)	100 (9)	0 (0)
LAP5/GFP		+		0 (0)	0 (0)	0 (0)	254 (17)	100 (6)	0 (0)
LAP6/GFP^e^		+		0 (0)	0 (0)	0 (0)	0 (0)	0.8 (1)	100 (14)
LAP6/GFP		+		0 (0)	0 (0)	0 (0)	0 (0)	0 (0)	100 (4)
									
LAP3/GFP (LAP1-KO)^g^		+		0 (0)	550 (27)	100 (18)	0 (0)	0 (0)	0 (0)
LAP3/GFP (LAP1-KO)		+		0 (0)	74 (12)	100 (12)	0 (0)	0 (0)	0 (0)
									
LAP3/GFP		+	+	270 (44)	282 (54)	100 (29)	222 (37)	151 (23)	15 (11)
LAP3/GFP		+	+	152 (52)	391 (84)	100 (50)	80 (35)	83 (24)	2.1 (12)
LAP3/GFP (LAP1-KO)		+	+	0 (0)	248 (56)	100 (32)	2.5 (4)	1.0 (3)	0 (0)
LAP1ΔSRCR/GFP^d^		+	+	54 (27)	322 (59)	100 (37)	2.9 (5)	1.0 (5)	0 (0)
LAP1ΔPTX/GFP^h^		+	+	112 (24)	216 (42)	100 (25)	56 (18)	18 (9)	1.8 (2)
									
LAP3ΔLCCL/GFP^c^		+		207 (59)	309 (79)	100 (42)	0 (0)	0 (1)	0 (0)
LAP3ΔLCCL/GFP		+	+	172 (49)	352 (75)	100 (32)	67 (35)	42 (24)	4.0 (11)

a Duplicate samples correspond to independent biological replicates.

b [11].

c [10].

d [5].

e [6].

f [16].

g [17].

h This paper (Fig. S1).

To further investigate LAP family member interactions, we carried out similar pull down experiments with parasite lines LAP4/GFP, LAP5/GFP and LAP6/GFP [16]. Transcripts of *lap4*, *lap5* and *lap6* are translationally repressed in gametocytes and not expressed as protein until after fertilization [16], therefore ookinete samples were used. Pull-down samples from purified LAP4/GFP or LAP5/GFP ookinetes contained high levels of LAP4 and LAP5, but little or no LAP1, LAP2, LAP3 or LAP6 (Tables 1, S1), showing that LAP4 and LAP5 interact with high avidity with each other, but not with the other LAPs. In pull-down samples of LAP6/GFP ookinetes, only LAP6 was detected in discernible amounts (Tables 1, S1), indicating that LAP6 does not bind to its family members with high avidity.

Based on domain topologies, LAP4 is considered a structural paralogue of LAP2, and LAP5 is a structural paralogue of LAP3 (Fig. 1). The interaction observed between LAP4 and LAP5 could therefore resemble that between LAP2 and LAP3. To test whether LAP2 could interact directly with LAP3, as shown for LAP4 and LAP5 (Tables 1, S1), we used a parasite line which expresses GFP-tagged LAP3 in a LAP1 knockout background [17]. GFP pull down samples from these parasites contained no LAP1, as expected, but still contained abundant LAP2 and LAP3 (Tables 1, S1). This shows that LAP2 and LAP3 interact with high avidity in the absence of LAP1.

The failure to pull down LAP4, LAP5 and LAP6 with GFP-tagged LAP1 or LAP3, and *vice versa* (Tables 1, S1), could reflect weak interactions that were lost during the cell lysis step before pull down. To test this hypothesis, ookinetes purified from parasite line LAP3/GFP were crosslinked *in vivo* by formaldehyde treatment before cell lysis (see supplemental Materials and Methods section). Formaldehyde is one of the shortest available cross-linkers is (2.3–2.7 Å) and low concentrations (0.4–2%) and short reaction times (minutes instead of hours) allow its utilization as a crosslinker to analyze protein–protein interactions [18]. Indeed, crosslinking resulted in the pull down of all six LAPs (Tables 1, S1), indicating that LAP4, LAP5 and LAP6 are part of the LAP complex albeit through weaker interactions, and join the LAP1/2/3 subcomplex after fertilization.

Given the structural and interaction similarities between the LAP2/3 and LAP4/5 pairs, we next hypothesized that the recruitment of the other LAPs to the complex could be mediated by LAP1. This was tested using ookinetes from the mutant parasite line that expresses LAP3::GFP in a LAP1 knockout background [17] combined with *in vivo* crosslinking. Using this approach, LAP4 and LAP5 were pulled down with markedly reduced efficacy relative to LAP2 and LAP3, while LAP6 failed to co-purify altogether (Tables 1, S1). These results indicate that LAP1 plays a key role in recruiting LAP4, LAP5 and LAP6 to the LAP complex.

To further dissect the role of LAP1 in these interactions, we carried out pull downs from ookinete lysates (after crosslinking) with parasite line LAP1ΔSRCR/GFP expressing LAP1::GFP without its two SRCR domains [5]. Removal of the SRCR domains of LAP1 results in a LAP1 knockout phenotype characterized by a lack of crystalloid biogenesis and sporozoite formation [5]. Pull down with this parasite gave a similar outcome as using parasites lacking LAP1 altogether: low amounts of LAP4 and LAP5 were pulled down relative to LAP1, LAP2 and LAP3, while LAP6 failed to co-purify (Tables 1, S1), indicating that the SRCR domains play a central role in LAP complex formation. The ability to efficiently pull down LAP2 and LAP3 in this experiment shows that the mutant LAP1 protein without the SRCR modules retains interaction with LAP2 and LAP3. LAP1 is the only protein in the genus *Plasmodium* that contains SRCR domains and, importantly, these domains are unique among all SRCR domains in possessing two additional cysteine residues in one of hypervariable loop-out regions between sheets β4 and β5 [6]. In CD6, hypervariable regions in the SRCR domain are involved in substrate interaction and specificity [19]. The unique cysteines contained in this hypervariable region could thus be involved in LAP1 interaction and function, for example by forming new intra-domain disulfides, or by interacting with other domains within LAP1 or other family members.

As an internal control for the LAP1ΔSRCR/GFP parasite we also generated a new parasite line named LAP1ΔPTX/GFP, which expresses LAP1::GFP without its pentaxin (PTX) domain (see supplemental Materials and Methods section and supplemental Fig. S1). Like LAP1ΔSRCR/GFP parasites, LAP1ΔPTX/GFP parasites failed to form crystalloids and generated oocysts, the large majority of which failed to produce sporozoites (Fig. S1). However, compared to LAP1ΔSRCR/GFP, pull down from LAP1ΔPTX/GFP ookinetes yielded considerably higher levels of LAP4, LAP5 and LAP6, albeit the amounts were reduced compared to ookinetes expressing the full-length LAP1 (Tables 1, S1). These observations indicate that the SRCR and PTX modules of LAP1 contribute differentially to the formation of the complete LAP complex. Moreover, the very similar phenotypes of these LAP1 mutant parasites indicate that the relative amounts of individual LAPs within the complex is equally important for its function.

Ablation or mutation of LAP1 did not appear to have a significant effect on the relative levels of LAP2 and LAP3, as opposed to LAP4, LAP5 and LAP6 (Tables 1, S1). The reduced levels of LAP4, LAP5 and LAP6 in these pull-down samples could reflect a reduced ability to bind to the gametocyte-specific LAP subcomplex. It is also possible that the stability of these proteins was adversely affected, possibly a direct consequence of sub-optimal binding to the other LAPs resulting in conformational changes and misfolding [17]. In this context it is important to note that in *P. falciparum* ablation of certain LAPs can adversely affect the level of other LAPs, a phenomenon that was called co-dependent expression [15]. Whatever the precise underlying mechanism, LAP1 clearly has a key role in formation of a complete and fully functional LAP complex.

Pull down samples from ookinetes of parasite line LAP3ΔLCCL/GFP, which expresses a version of LAP3::GFP that lacks its LCCL domain (previously described as *Pb*LAP3/LCCL-KO [10]) contained high levels of LAP1, LAP2 and LAP3 similar to full-length LAP3/GFP pull-downs (Tables 1, S1). Likewise, *in vivo* crosslinked ookinete samples of parasite line LAP3ΔLCCL/GFP gave rise to co-purification of all LAP family members similar to full-length LAP3/GFP pull-downs (Tables 1, S1). These combined data show that the LCCL domain of LAP3 is not required for the formation of the LAP1/2/3 sub-complex or indeed the complete LAP complex, indicating that this LCCL domain is not significantly involved in the LAP interactions. This is consistent with the observation that mature ookinetes of this parasite can form crystalloids and give rise to normal sporozoite development and transmission [10]. However, crystalloid biogenesis in LAP3ΔLCCL/GFP ookinetes is retarded [10], which suggests that the LCCL domain of LAP3 does nonetheless have a subtle role within the LAP complex enhancing downstream crystalloid genesis.

Whilst recognizing the limitations of *in vitro* interaction studies with bacterially expressed proteins compared to parasite-expressed equivalents (e.g. with regards to protein conformation), the LAP interactions reported for *P. falciparum* [15] are broadly consistent with this study and point to a conservation of LAP interactions between the two *Plasmodium* species. This concept is strongly supported by the highly conserved and unique architectures of the LAP family members. It is important to note that in *P. falciparum* all six LAP homologues are expressed as protein in gametocytes [20], in contrast to *P. berghei* where transcripts of *lap4, lap5* and *lap6* are translationally repressed in gametocytes resulting in their protein expression post-fertilization [16]. The different strength interactions between different LAP combinations as identified in this study could be a mechanism to ensure that complex formation of the LAP homologues in *P. falciparum* follows a similar order of events to that in *P. berghei* despite all LAPs being present at the same time. Furthermore, gametocyte development in *P. falciparum* takes much longer than in *P. berghei,* increasing the likelihood that staggered LAP expression and complex assembly as shown here for *P. berghei* could occur in the human malaria parasite during gametocytogenesis.

Unravelling the molecular interactions of the LAP complex is important, because its disruption could be a way to achieve malaria transmission-blockade. LAPs are already expressed in blood stage gametocytes, particularly in *P. falciparum*, and accordingly the LAP complex could potentially be targeted in the human host before the parasite enters the mosquito. Furthermore, LAP knockout and mutational studies show that disruption of the LAP complex does not affect the parasite until after oocyst development, so the ookinete and oocyst burden in the insects are not reduced, yet the insects are not infective. Targeting the LAP complex therefore has the advantages that it would not rely on the uptake of the active compounds by the vector mosquito, and it would minimize risk of enhancing mosquito fitness as a consequence of lowering the parasite load in the insect, which could enhance vectorial capacity [21]. The identification of LAP1, and particularly of its SRCR domains, as critical sites for LAP interaction and complex formation will aid future design and identification of small molecule inhibitors of these processes and downstream parasite transmission.
