# Supplementary material for: LCCL protein complex formation in Plasmodium is critically dependent on LAP1
Source: Mol Biochem Parasitol. 2017 Jun;214:87–90. doi: 10.1016/j.molbiopara.2017.04.005 (PMC5482319; doi:10.1016/j.molbiopara.2017.04.005)
Supplement: Supplementary file 1 [file mmc1.pdf]

LCCL protein complex formation in Plasmodium is critically dependent on LAP1

Annie Z. Tremp, Vikram Sharma, Victoria Carter, Edwin Lasonder and Johannes T. Dessens

Supplemental Materials and Methods section

Laboratory animal use

All laboratory animal work is subject to regular ethical review by the London School of Hygiene and Tropical Medicine, and has approval from the United Kingdom Home Office. Work was carried out in accordance with the United Kingdom Animals (Scientific Procedures) Act 1986 implementing European Directive 2010/63 for the protection of animals used for experimental purposes. Experiments were conducted in 6-8 weeks old female CD1 mice, specific pathogen free and maintained in filter cages. Animal welfare was assessed daily and animals were humanely killed upon reaching experimental or humane endpoints. Mice were infected with parasites by intraperitoneal injection, or by infected mosquito bite on anaesthetized animals. Parasitemia was monitored regularly by collecting of a small drop of blood from a superficial tail vein. Drugs were administered by intraperitoneal injection or where possible were supplied in drinking water. Parasitized blood was harvested by cardiac bleed under general anaesthesia without recovery.

Parasite maintenance, transmission, culture and purification

P. berghei ANKA clone 2.34 parasites were maintained as cryopreserved stabilates or by mechanical blood passage and regular mosquito transmission. Mosquito infection and transmission assays were as previously described using Anopheles stephensi [1, 2] and infected insects were maintained at 20°C at approximately 70% relative humidity under a 12h/12h light/dark cycle. Ookinete cultures were set up overnight from gametocytemic blood as previously described [3]. After 20-24h, ookinetes were purified by ice-cold 0.17M ammonium chloride lysis and centrifugation at 800×*g* for 10 min, followed by three washes in phosphate buffered saline (PBS). Gametocytes were purified as described [4].

Generation of parasite line LAP1ΔPTX/GFP

To study the role of the pentaxin (PTX) domain of LAP1 we generated by allelic replacement a parasite line expressing LAP1::GFP without its PTX domain, named LAP1ΔPTX/GFP (Fig. S1A). One µg of plasmid pDNR-PbSR/EGFP [5] served as template in PCR using primers deltaPTX-F (GGCTAGCTGATGGTATGTCTGGAAATAGAGACATAAATAC) and deltaPTX-R (ATACCATCAG-CTAGCCATTGAAAGTTGGTTC). After PCR amplification (1 cycle of 94º for 2min; 5 cycles of 94ºC for 30sec, 62ºC for 8min), the template DNA was digested with DpnI and the amplicon DNA circularized via In-Fusion PCR cloning to give plasmid pDNR-deltaPTX/EGFP. The LAP1-specific insert contained within pDNR-deltaPTX/EGFP was then introduced into pLP-DHFR/SR [5] via Cre-LoxP site-specific recombination to give the transfection vector pLP-deltaPTX/EGFP. Prior to performing transfections, plasmid DNA was digested with KpnI and SacII to remove the vector backbone. Parasite transfection,

pyrimethamine selection and dilution cloning were performed as previously described [6]. Genomic DNA extraction was performed as previously described [2].

Immuno-affinity capture and in vivo crosslinking

Immuno-affinity capture of GFP fusion proteins was carried out by 'GFP pull-down' using the μMACS GFP tagged protein isolation kit (Miltenyi Biotec) according to manufacturer's instructions. Briefly, 5-10 million purified parasites were lysed in 1ml of pre-cooled lysis buffer (150mM NaCl, 1% Triton X-100, 50mM Tris-HCl pH8.0) and incubated on ice for 30min. Cell debris was removed by centrifugation at 10,000xg. Fifty μl anti-GFP microbeads were added to the supernatant and the mixture incubated on ice for 30min to allow antibody binding. The cell lysate was then run by gravity through a magnetic microcolumn to capture the magnetic microbeads, followed by four 200μl washes with lysis buffer. Proteins were eluted in 50μl pre-heated 95°C elution buffer (50mM Tris-HCl pH6.8, 50mM DTT, 1% SDS, 1mM EDTA, 0.005% bromophenol blue, 10% glycerol) and frozen until further use.

For *in vivo* crosslinking, purified ookinetes were collected by low speed centrifugation (0.8 x g), resuspended in 0.5ml PBS supplemented with 1% (w/v) paraformaldehyde and incubated at room temperature. The cells were collected by centrifugation after a total of 10min in the fixative (including centrifugation), resuspended in 0.5ml 250mM Tris-HCl (pH 7.2), and incubated 10min at room temperature to quench the formaldehyde. Cells were again collected by centrifugation followed by cell lysis and GFP pull-down as described above.

Sample preparation for mass spectrometry

Protein samples in SDS sample buffer were digested with trypsin by a modified version of the filter-aided sample preparation (FASP) procedure of in solution digestion [7]. Samples were reduced in sample buffer with 100mM DTT for 3 minutes at 95 before centrifuging them in Amicon Ultra filter tubes (30kDa cut off) for 15 minutes at 16000g and subsequent dilution of samples with 300ul of 50mM ABC buffer. Samples were centrifuged once more to ensure maximal removal of DTT and SDS followed by alkylation step with 50mM of 2-Chloroacetamide (Sigma). Single step overnight trypsin digestion at enzyme to substrate ratio of 1:100 was carried out at 37°C. Tryptic digests were acidified to a final concentration of 0.1% TFA and purified by STAGE tips [8].

Liquid chromatography tandem mass spectrometry

Peptide digest samples were analyzed by an LC-MS/MS platform composed of the Ultimate 3000 UPLC (Thermo Fisher, Germany) connected to the Orbitrap Velos Pro mass spectrometer (Thermo Fisher, Germany) for acquiring tandem mass spectrometry data. Peptide samples were loaded on a 2 cm Acclaim™ PepMap™100 Nano-Trap Column (Thermo Fisher, Germany) and were separated by a 25 cm Acclaim™ PepMap™100 Nano LC column (Thermo Fisher, Germany) packed with 3 μm C18 beads with a flow-rate of 300nl/min in a 120 min gradient of 95% buffer A/5% buffer B to 65% buffer A /35 % buffer B (buffer A: 0.5% acetic acid. Buffer B: 0.5% acetic acid in 100% acetonitrile). Peptides eluting from the column were

ionised and injected into the mass spectrometer at 2.3 kV pray voltage. The Orbitrap mass spectrometer operated in a data-dependent mode and switched between MS and MS2 automatically by a top 10 method. The Orbitrap cell acquired full-scan spectra of intact peptides (m/z 350-1500) with automated gain control accumulation value of 1.000.000 ion and with a resolution of 60.000. The ten most abundant ions were sequentially isolated and fragmented in the linear ion trap, where dissociation was induced through collision, using an accumulation target value of 10.000, a normalized collision energy of 35% and a capillary temperature of 275°C. Dynamic exclusion of ions sequenced within the 45 previous seconds was applied. Unassigned charge states and singly charged ions were excluded from sequencing. For MS2 selection, a minimum of 10.000 counts was required.

Protein identification and quantification

Tandem mass spectrometry data was searched by Andromeda [9] search engine integrated in MaxQuant (Version 1.5.3.8) [10] for protein identification. Peak lists were generated for the top 12 most intense MS peaks in 100 Da windows by MaxQuant prior to the database search. The protein database contained protein sequences from *P. berghei* (http://plasmodb.org/common/downloads/release-29/PbergheiANKA/fasta/data/) and from mouse (http://www.uniprot.org/downloads, downloaded at 2 November 2016) supplemented with frequently observed contaminants. Andromeda search parameters for protein identification were set to tolerance of 6 ppm for the parental peptide and 0.5 Da for fragmentation spectra and trypsin specificity allowing up to 2 miscleaved sites. Deamination of glutamine, oxidation of methionine, and asparagine and protein N-terminal acetylation were set as variable modifications, carboxyamidomethylation of cysteines was specified as a fixed modification. Minimal required peptide length was specified at 7 amino acids. Peptides and proteins detected by at least two peptides in one of the samples with a false discovery rate (FDR) of 1% were accepted. Excluded from validation were proteins identified by site only, external contaminants and reversed proteins. Proteins were quantified by normalized summed peptide intensities [11] computed in MaxQuant with the label free quantification (LFQ) option switched on.

References

[1] Khater EI, Sinden RE, Dessens JT. A malaria membrane skeletal protein is essential for normal morphogenesis, motility, and infectivity of sporozoites. J Cell Biol. 2004;167:425-32.

[2] Dessens JT, Beetsma AL, Dimopoulos G, Wengelnik K, Crisanti A, Kafatos FC, et al. CTRP is essential for mosquito infection by malaria ookinetes. EMBO J. 1999;18:6221-7.

[3] Arai M, Billker O, Morris HR, Panico M, Delcroix M, Dixon D, et al. Both mosquito-derived xanthurenic acid and a host blood-derived factor regulate gametogenesis of *Plasmodium* in the midgut of the mosquito. Mol Biochem Parasitol. 2001;116:17-24.

[4] Raabe AC, Billker O, Vial HJ, Wengelnik K. Quantitative assessment of DNA replication to monitor microgametogenesis in *Plasmodium berghei*. Mol Biochem Parasitol. 2009;168:172-6.

[5] Carter V, Shimizu S, Arai M, Dessens JT. PbSR is synthesized in macrogametocytes and involved in formation of the malaria crystalloids. Mol Microbiol. 2008;68:1560-9.

[6] Waters AP, Thomas AW, van Dijk MR, Janse CJ. Transfection of malaria parasites. Methods. 1997;13:134-47.

[7] Wisniewski JR, Zougman A, Nagaraj N, Mann M. Universal sample preparation method for proteome analysis. Nat Methods. 2009;6:359-62.

[8] Rappsilber J, Ishihama Y, Mann M. Stop and go extraction tips for matrix-assisted laser desorption/ionization, nanoelectrospray, and LC/MS sample pretreatment in proteomics. Anal Chem. 2003;75:663-70.
[9] Cox J, Neuhauser N, Michalski A, Scheltema RA, Olsen JV, Mann M. Andromeda: a peptide search engine integrated into the MaxQuant environment. J Proteome Res. 2011;10:1794-805.
[10] Cox J, Mann M. MaxQuant enables high peptide identification rates, individualized p.p.b.-range mass accuracies and proteome-wide protein quantification. Nat Biotechnol. 2008;26:1367-72.
[11] Cox J, Hein MY, Luber CA, Paron I, Nagaraj N, Mann M. Accurate proteome-wide label-free quantification by delayed normalization and maximal peptide ratio extraction, termed MaxLFQ. Mol Cell Proteomics. 2014;13:2513-26.
