# Supplementary material for: LCCL protein complex formation in Plasmodium is critically dependent on LAP1
Source: Mol Biochem Parasitol. 2017 Jun;214:87–90. doi: 10.1016/j.molbiopara.2017.04.005 (PMC5482319; doi:10.1016/j.molbiopara.2017.04.005)
Supplement: Supplementary file 2 [file mmc2.pdf]

LCCL protein complex formation in Plasmodium is critically dependent on LAP1

Annie Z. Tremp, Vikram Sharma, Victoria Carter, Edwin Lasonder and Johannes T. Dessens

Table S1. Relative abundance of LAPs in GFP pull down samples from *Plasmodium berghei* parasite lines.

Parasite line	gametocyte	ookinete	crosslinked	LFQ intensity						Total proteins[1] detected
				LAP1	LAP2	LAP3	LAP4	LAP5	LAP6	
LAP3/GFP	+			76001000	78178000	33188000	0	0	0	146
LAP3/GFP	+			57151000	19142000	20605000	0	0	0	53
LAP3-KO	+			0	0	0	0	0	0	65
LAP3/GFP		+		141100000	147900000	56656000	84248	0	0	151
LAP3/GFP		+		208560000	203210000	46269000	278540	0	0	145
LAP3-KO		+		0	0	0	0	0	0	111
LAP1/GFP		+		77120000	22450000	7093800	0	0	0	46
LAP1-KO		+		0	0	0	0	0	0	38
LAP4/GFP		+		0	0	0	14160000	23920000	317420	123
LAP4/GFP		+		0	0	0	41938000	7493700	0	53
LAP5/GFP		+		0	0	0	23050000	3532700	0	138
LAP5/GFP		+		0	0	0	12929000	5087900	0	140
LAP6/GFP		+		0	0	0	0	32435	4041100	127
LAP6/GFP		+		0	0	0	0	0	783480	73
LAP3/GFP (LAP1-KO)		+		0	107610000	19640000	0	0	0	32
LAP3/GFP (LAP1-KO)		+		0	2968900	4020400	0	0	0	71
LAP3/GFP		+	+	80922000	84715000	30014000	66883000	45241000	4486800	138
LAP3/GFP		+	+	132900000	341900000	87438000	70305000	72480000	1849900	117
LAP3/GFP (LAP1-KO)		+	+	0	77154000	31079000	783880	252280	0	167
LAP1ΔSRCR/GFP		+	+	31100000	183590000	57184000	1670300	554360	0	98
LAP1ΔPTX/GFP		+	+	30212000	58171000	26993000	15206000	4767100	493400	83
LAP3ΔLCCL/GFP		+		163040000	243100000	78705000	0	0	0	122
LAP3ΔLCCL/GFP		+	+	69466000	142360000	40358000	27266000	16924000	1624200	167

[1] Includes both parasite and mouse proteins (1% False Discovery Rate).
