# Supplementary material for: LCCL protein complex formation in Plasmodium is critically dependent on LAP1
Source: Mol Biochem Parasitol. 2017 Jun;214:87–90. doi: 10.1016/j.molbiopara.2017.04.005 (PMC5482319; doi:10.1016/j.molbiopara.2017.04.005)
Supplement: Supplementary file 3 [file mmc3.pdf]

*LCCL protein complex formation in Plasmodium is critically dependent on LAP1*

*Annie Z. Tremp, Vikram Sharma, Victoria Carter, Edwin Lasonder and Johannes T. Dessens*

**Supplemental Figure S1**

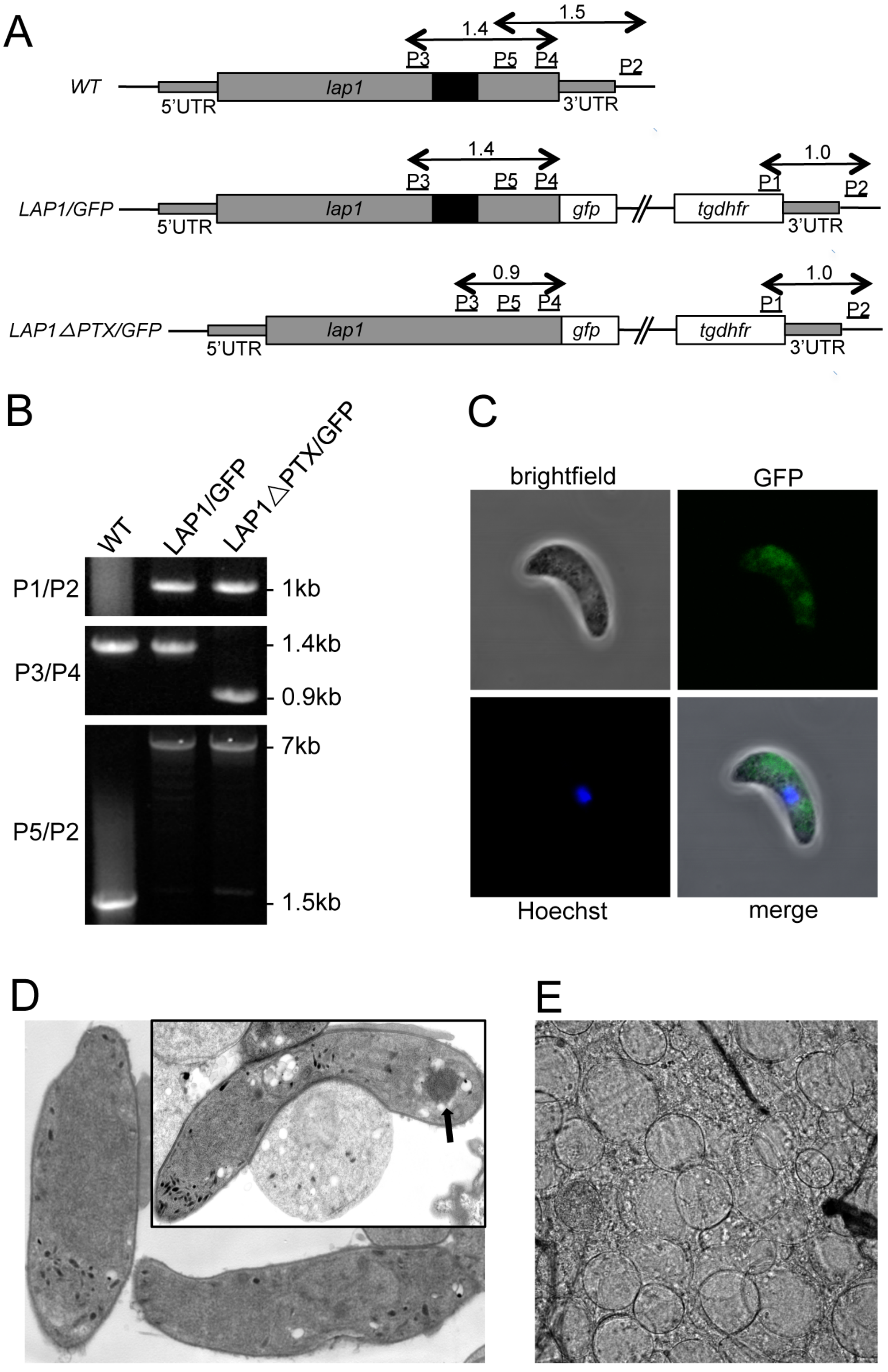

**Fig. S1** Genotypic and phenotypic analyses of parasite line LAP1ΔPTX/GFP. **A**: Structure of wildtype (WT) and modified *lap1* alleles in parasite lines LAP1/GFP and LAP1ΔPTX/GFP after double crossover homologous recombination. The *lap1* gene is indicated with coding sequence (wide grey bars) and 5' and 3' untranslated regions (UTRs) (narrow grey bars). Also indicated are the PTX domain (black); the GFP module; the *Tg*DHFR selectable marker gene cassette and primers used for diagnostic PCR amplification (P1-P5). Primer P2 sequence is not present within the targeting vector. Diagnostic PCR product sizes are indicated with arrowed lines and numbers (kb). **B**: Diagnostic PCR across the 3'-integration site with primers P1 (TCGTGGGCTACGTCCCGCAC) and P2 (CGCCTTCACGCTGATGT) amplify an approximately 1kb product in parasite lines LAP1ΔPTX/GFP as well as in parasites expressing full-length GFP-tagged LAP1, showing integration of the selectable marker into the *lap1* locus. Diagnostic PCR with primers P3 (AAACATTTTTCGAGCATAATATG) and P4 (ATGAGGGCCCCTAAGCTTAAGCGTTTCAAAAAGG-TAAATGA) amplify a 1.4kb product from both WT parasites and parasites expressing full-length GFP-tagged LAP1, while they amplify a 0.9kb fragment from parasite line LAP1ΔPTX/GFP, confirming absence of the PTX domain-encoding sequence in the latter parasite. Diagnostic PCR with primers P5 (GCATGATGAGGATAATAATAAAACT) and P2 amplify an approximately 1.5kb fragment only from the parental WT parasites, confirming absence of the unmodified *lap1* allele in the transgenic parasite lines. The same primer pair instead amplify much larger products in the transgenic parasite lines (approximately 7.7kb and 7.2kb in LAP1/GFP and LAP1ΔPTX/GFP, respectively), because of the presence of additional sequences between the primer annealing sites. **C**: Confocal brightfield and fluorescence images of a typical ookinete of parasite line LAP1ΔPTX/GFP, showing absence of focal spots corresponding to crystalloids. **D**: Transmission electron micrograph of LAP1ΔPTX/GFP ookinetes, showing absence of crystalloids. Inset shows an equivalent image of a wildtype ookinete with crystalloid (arrow). **E**: Brightfield image of an *Anopheles stephensi* midgut section at 2 weeks post-infection with parasite line LAP1ΔPTX/GFP, showing non-sporulating oocysts.
